# Mass spectrometry-based determination of Kallikrein-related peptidase 7 (KLK7) cleavage preferences and subsite dependency

**DOI:** 10.1038/s41598-017-06680-4

**Published:** 2017-07-28

**Authors:** Lakmali Munasinghage Silva, Thomas Stoll, Thomas Kryza, Carson Ryan Stephens, Marcus Lachlan Hastie, Helen Frances Irving-Rodgers, Ying Dong, Jeffrey John Gorman, Judith Ann Clements

**Affiliations:** 1 Queensland University of Technology (QUT), Institute of Health and Biomedical Innovation (IHBI) and School of Biomedical Sciences at the Translational Research Institute, 37 Kent Street, Woolloongabba, Queensland 4102 Australia; 2 QIMR Berghofer Medical Research Institute, 300 Herston Road, Herston, Queensland 4006 Australia; 3 School of Medical Science, Griffith University Gold Coast Campus, Parklands Drive, Southport, Queensland 4215 Australia; 4 Present Address: Proteases and Tissue Remodelling Section, National Institute of Dental and Craniofacial Research, National Institutes of Science, 30 Convent Drive, Bethesda, Maryland 20892 USA

## Abstract

The cleavage preferences of Kallikrein-related peptidase 7 (KLK7) have previously been delineated using synthetic peptide libraries of fixed length, or single protein chains and have suggested that KLK7 exerts a chymotryptic-like cleavage preference. Due to the short length of the peptides utilised, only a limited number of subsites have however been assessed. To determine the subsite preferences of KLK7 in a global setting, we used a mass spectrometry (MS)-based in-depth proteomics approach that utilises human proteome-derived peptide libraries of varying length, termed Proteomic Identification of protease Cleavage Sites (PICS). Consistent with previous findings, KLK7 was found to exert chymotryptic-like cleavage preferences. KLK7 subsite preferences were also characterised in the P2-P2′ region, demonstrating a preference for hydrophobic residues in the non-prime and hydrophilic residues in the prime subsites. Interestingly, single catalytic triad mutant KLK7 (mKLK7; S195A) also showed residual catalytic activity (k_cat_/K_M_ = 7.93 × 10^2^ s^−1^M^−1^). Catalytic inactivity of KLK7 was however achieved by additional mutation in this region (D102N). In addition to characterising the cleavage preferences of KLK7, our data thereby also suggests that the use of double catalytic triad mutants should be employed as more appropriate negative controls in future investigations of KLK7, especially when highly sensitive MS-based approaches are employed.

## Introduction

Human Kallikrein-related peptidase 7 (KLK7) is a member of a family composed of 15 serine peptidases clustered on chromosome 19q13.4^1–3^ and reported to be involved in many pathological conditions, including skin inflammation, ovarian and many other cancers^4–8^, although its mechanism of action is still not known. Detailed analysis of its substrate specificity is essential in determining its natural substrate repertoire, to guide in designing inhibitors, artificial peptide substrates and subsequently in determining its functional consequences. To date, only a small number of studies have been performed^9–11^, where the prime (S’) and non-prime (S) side of the scissile peptide bond (Schechter and Berger nomenclature^12^) have been studied independently.

To further characterise the substrate specificity of KLK7, we employed a peptide-centric proteomics approach, which utilises human proteome-derived peptide libraries and liquid chromatography coupled with tandem mass spectrometry (LC-MS/MS), termed Proteomic Identification of protease Cleavage Sites (PICS)^13^ to determine KLK7 substrate preferences on both the prime and non-prime sides simultaneously. Thereby, we determined that KLK7 shows chymotryptic-like cleavage specificity by cleaving C-terminal to hydrophobic amino acids, tyrosine (Y), leucine (L), phenylalanine (F), methionine (M) and tryptophan (W) at the P1 position, in agreement with previous studies^9–11^. Furthermore, KLK7 S2 and S3 subsites showed selectivity for hydrophobic amino acids, such as leucine (L) and valine (V) at the S2 and alanine (A) at the S3 subsite. Nonetheless, the prime side, S2′ and S3′ subsites, showed specificity to hydrophilic amino acid lysine (K) and a hydrophobic amino acid, alanine (A) to a lesser extent. This study is the first study to determine KLK7 cleavage specificities in a more global manner, analyzing subsite preferences of KLK7 on both prime and non-prime sites simultaneously.

As part of our research into the biological function of the KLK7 peptidase, we sought to ensure that the mutant peptidases we had generated were indeed catalytically inactive. Although mutant KLK7 (mKLK7) carrying the single mutation (S195A) in the catalytic triad has been widely used in *in vitro* cell-based protease research^14^, the activity has not been measured using more highly sensitive in-depth proteomics approaches. We observed that the single mKLK7 has residual chymotryptic-like activity by cleaving C-terminal to Tyrosine (Y), Leucine (L) and Phenylalanine (F) at the P1 position in line with active KLK7 (k_cat_/K_M_ = 7.93 × 10^2^ s^−1^M^−1^). High sequence and/or structural similarity is evident between the KLK7 and KLK4 and KLK5 proteins, suggesting potential residual activity may also occur in S195A mutant KLK4 and KLK5. We then produced S195A mKLK4, which also showed residual catalytic activity (k_cat_/K_M_ = 6.13 × 10^3^ s^−1^M^−1^). These observations of residual catalytic activity of single mKLK4 and mKLK7 when mutated at the Ser residue led us to make an additional point mutation in both the mKLK7 and mKLK4 catalytic triad, D102N (asparagine), which showed no catalytic activity in subsequent assays.

## Results

### Single mutant KLK7 does not appear to have any residual activity

**Figure 1:**
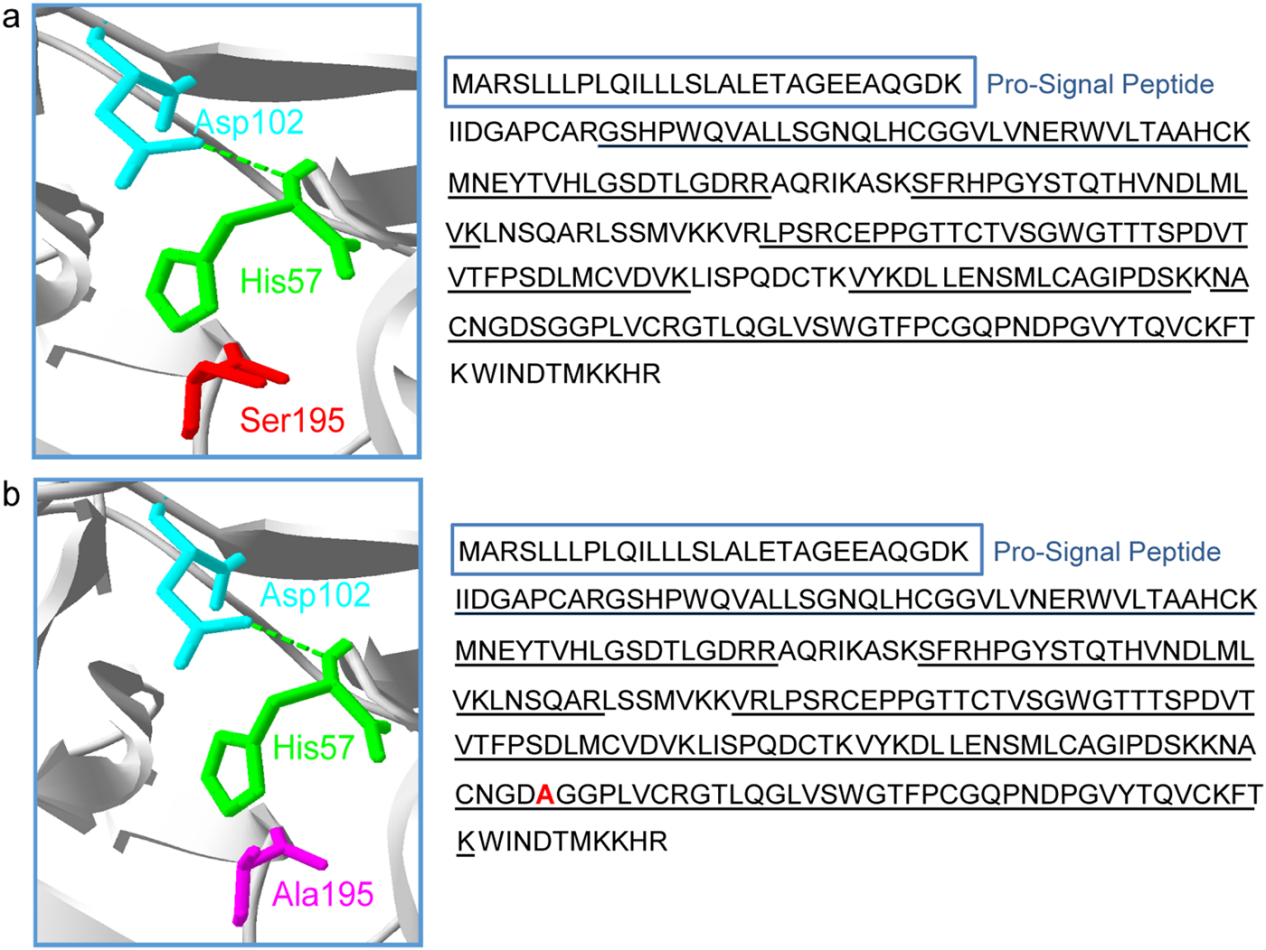
KLK7 and mKLK7 protein identity. (**a**) Surface exposed residues corresponding to the catalytic triad (His57, Asp102 and Ser195; Schechter and Berger notation^12^) of KLK7 were identified on the three dimensional (3D) structure available in Protein Data Bank (PDB, accession: 2QXG.pdb in standard serine protease orientation) using SPDBV v4.10. (**b**) In addition, S195A mutation was made using the mutation tool on SPDBV v4.10. Alongside, are the peptides identified in the MS analysis (Underlined; identified with a 99% protein probability and 95% peptide probability cut off) of trypsin-digested (**a**) active KLK7 and (**b**) mKLK7 aligned with the KLK7 full length protein (UniProtKB; P49862-1). The pro-signal peptide (in blue box) was not included in the expression construct, thus obtaining mature amino acid sequences for these peptidases. Expected mutation, S195A was identified in the MS-identified mKLK7 peptide sequence as highlighted in red bold letter.

Active KLK7 and mKLK7 (S195A) were produced in the *Pichia pastoris* expression system. To ensure correct sequences with appropriate mutations where expected, sequence coverage analysis of the produced peptidases was performed using trypsin digestion followed by MS analysis. Only the peptides identified with high confidence (i.e. >99% protein and >95% peptide probability) were further analysed (Supplementary Table 1). Recombinant active KLK7 was identified with 12 unique peptides, 24 unique spectra (out of a total of 32) and 68% protein coverage (172/253 residues) (Fig. 1a). Similarly, recombinant mKLK7 was identified with 21 unique peptides, 49 unique spectra and 79% protein coverage (199/253 residues). High confidence tryptic peptides were identified with the S195A mutation confirming successful generation of mKLK7 (Fig. 1b). Moreover, minimal protein contamination from the expression system was identified.

**Figure 2:**
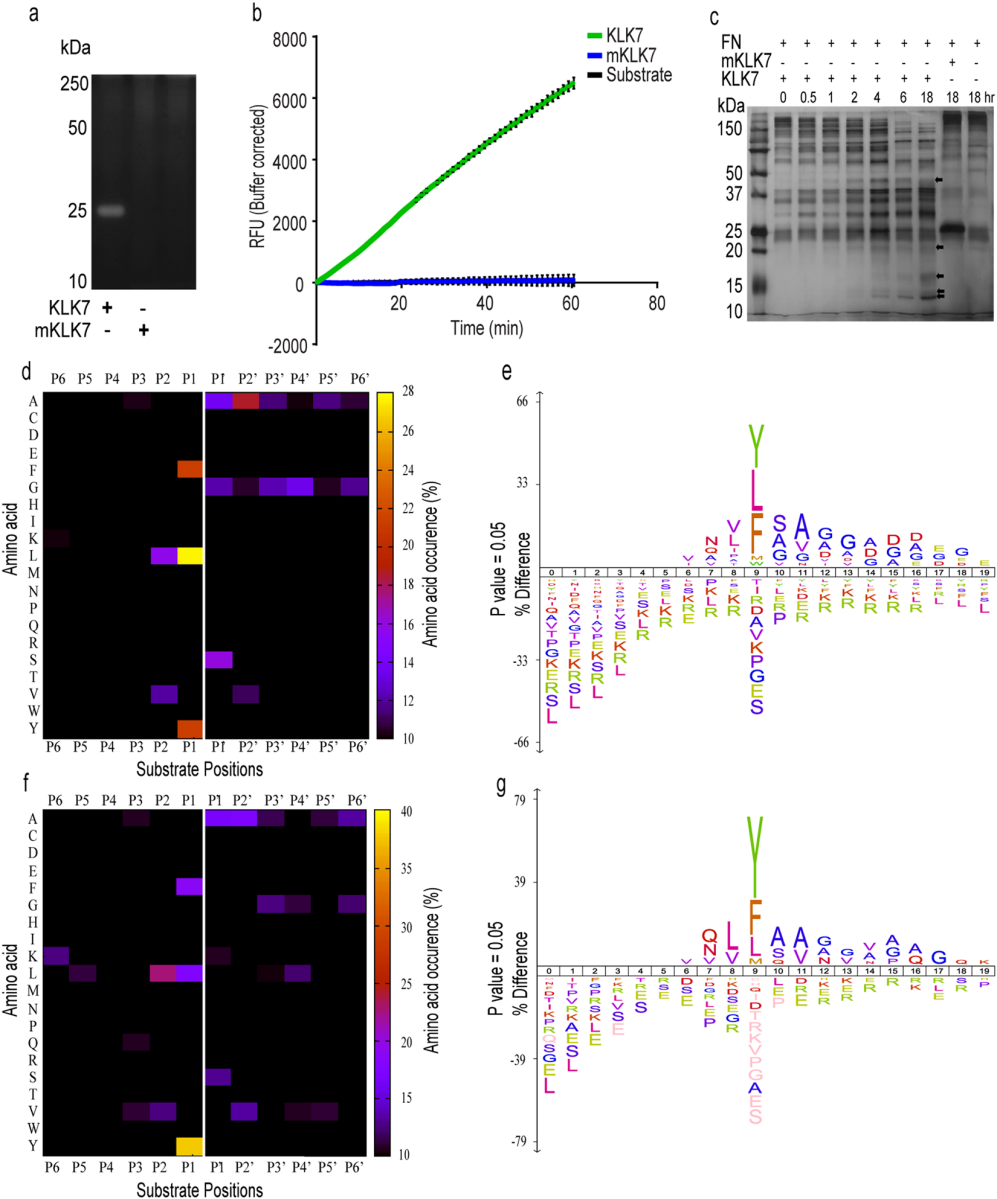
Activity of KLK7 and mKLK7. (**a**) Coomassie-stained 1% casein zymography: cleared area at approximately 25 kDa shows digestion of casein by active KLK7 while mKLK7 has not digested casein, suggesting no activity. (**b**) Activity with a KLK7-specific peptide substrate MeO-Suc-Arg-Pro-Tyr-MCA: time versus absorbance (corrected mean ± SD) plot showing activity of KLK7 compared to no activity with mKLK7 and substrate only control (n = 6; mean ± SD). (**c**) Activity against a KLK7 protein substrate fibronectin: KLK7 and mKLK7 incubated with fibronectin (KLK7/FN: 1/10 molar ratio) visualised by silver-stained SDS-PAGE. Black arrow heads indicate KLK7-generated cleavage fragments. No cleavage was observed for mKLK7. (**d**) For KLK7 and (**f**) mKLK7 the percentage amino acid occurrences in P6-P6′ derived from both libraries were calculated and are shown in the form of a two-dimensional heat map. Both libraries confirm the predominant KLK7 specificity for Y, L and F in P1. The mKLK7-treated (**f**) tryptic and GluC libraries confirm the mKLK7 specificity for Y in P1. White line depicts the scissile peptide bond between P1 and P1′. (**e**) KLK7 and (**g**) mKLK7 cleavage sites derived from both libraries are displayed as icelogos by analysing their multiple sequence alignments. The height of the single amino acid residue (in bits) reflects its occurrence rate for each position in P10-P10′. Amino acids are coloured according to their physico-chemical properties.

Activity of these peptidases was first analysed using casein-zymography, a KLK7-specific peptide substrate and a protein substrate, fibronectin (FN). Only the active KLK7 but not mKLK7 showed proteolytic activity on the casein-zymogram as indicated by a cleared area at ~25 kDa (Fig. 2a). Activity is further indicated by increasing absorbance over time towards the MeO-Suc-Arg-Pro-Tyr-MCA peptide substrate (Fig. 2b) and cleavage of the protein substrate, fibronectin (Fig. 2c); mKLK7 appeared to have no activity.

### The Proteomic Identification of protease Cleavage Sites (PICS) analysis of KLK7

To assess the cleavage preferences of KLK7, PICS analysis was performed by first generating tryptic and GluC peptide libraries by treating HEK-293 whole cell lysate with either trypsin (cleaves C-terminal to Arg/Lys) or GluC (cleaves C-terminal to Glu/Asp) enzymes. The generated Tryptic and GluC libraries were treated with equivalent amounts of KLK7 or mKLK7 peptidase followed by the isolation of neo-N-termini and analysis by LC-MS/MS. The active KLK7 cleavage site specificities were predominantly chymotryptic-like by cleaving C-terminal to Y, L, F, M and W at the P1 position (Fig. 2d,e). Additionally, the other significant KLK7 cleavage site specificities, natural abundance >2, besides the L, F, Y, M and W at the P1 position, were primarily located in P2-P2′ (Supplementary Figure 1). Outside of P2-P2′, except glycine (G) at the P3′-P6′ positions, the amino acid ratios did not significantly exceed the natural amino acid composition, suggesting the non-random occurrence of amino acid levels in P2-P2′ (Supplementary Figure 1). Therefore, the frequency of occurrence of each unique P2-P2′ sequence combination was assessed.

In the KLK7-cleaved tryptic library, 130 P2-P2′ sequences were repeatedly identified in different peptides and L, F, Y, M and W were present in 28.5%, 23.1%, 21.7%, 5% and 4.1% of all P1 positions (1403) respectively. Similarly, in the GluC library, 20 P2-P2′ sequences were repeated in different peptides and L, Y, F, R, M and W were present in 25.8%, 20.4%, 15%, 6.5%, 4.5% and 2.3% of all P1 positions (353) respectively (Supplementary Figure 2). As shown previously^15^, KLK7 was detected to cleave C-terminal to R at the P1 position to a much lesser extent (6.5% of all cleavages detected), confirming minor tryptic-like specificities of KLK7.

In addition, analysis of the P2-P2′ peptides revealed subsite cooperativity of KLK7. In this analysis only the GluC-derived peptides were analysed as this library allowed for screening of both tryptic- and chymotryptic-like specificities (Supplementary Figure 2b). Hydrophobic amino acids, predominantly L and V were preferred at the P2 position, as shown previously^16^. Interestingly, the oxidized insulin β chain cleavage site, Glu-Ala-Leu-Tyr^**↓**^Leu-Val-Cya-Gly (^↓^indicates cleavage) matched with the above cleavage profile. On the other hand, hydrophilic K was preferred at the P1′ position in agreement with the cathelicidin antimicrobial peptide, Gly-Lys-Glu-Phe^**↓**^Lys-Arg-Ile-Val^17^ and the P2′ position showed selectivity to hydrophobic A and V predominantly and hydrophilic K to a lesser extent. Moreover, subsite dependency between P3-P3′ for all positional occurrences >1 × natural abundance was calculated using the CLIP-PICS web server^18^ (Table 1). Therein, occurrence of A, K, N, Q and V at the P3 position was shown to positively affect amino acid occurrence at P2, P1′ and P3′ positions and vice versa.

**Table 1:** Potential subsite cooperativity analysis for KLK7.

Fixed residue	Affected residue(s)	Change (percentage-points)	Vice-Versa change
P3_A	P1prime_A	11.6	11.4
P3_A	P2_V	**16.4**	**16**
P3_K	P2_K	11.5	12.5
P3_N	P1prime_A	**18.6**	11.2
P3_Q	P1prime_R	13.4	12.4
P3_Q	P2_K	10.8	12.2
P3_Q	P3prime_D	**17.1**	14.6
P3_Q	P3prime_V	**18.2**	11.1
P3_V	P2_P	**22**	14.9
P3_V	P3prime_N	10.6	**19.1**
P2_K	P1prime_S	**19.3**	12.3
P2_K	P2prime_N	14.8	**20.8**
P2_V	P1_F	−12.7	−10.3
P2_V	P1prime_A	11.1	11.1
P1_Q	P3prime_I	**15.6**	11.3
P1prime_H	P2prime_M	**21.3**	**19.7**
P1prime_R	P3prime_V	**15.9**	10.5
P2prime_N	P3prime_I	**25.1**	13

Potential subsite cooperativity analysis for 353 KLK7 cleavage sites detected in the GluC library was calculated using the CLIP-PICS web server (http://clipserve.clip.ubc.ca/pics)^18^. Minimum difference for subsite dependency was set to ±10 percentage-points. Subsite dependency was checked for all positional occurrences >1 × natural abundance and restricted to P3-P3′. Subsites with a change ≥15 percentage-point are bold.

### The PICS analysis of mKLK7 suggests mKLK7 still has residual activity

Intriguingly, mKLK7-treated tryptic and GluC peptide libraries showed specificity to Y, F and to a lesser extent L, at the P1 position (Fig. 2f,g), as per those treated with KLK7. Similarly, in mKLK7-treated samples, additional significant cleavage site specificities were located between P2-P2′ while occurrence of other amino acids in other positions did not significantly exceed natural occurrence. These observations therefore suggested that mKLK7 (S195A) retains residual enzymatic activity. Overall, 121 mKLK7-cleaved peptides identified in the tryptic library, with 119 peptides possessing different P2-P2′ sequences (Supplementary Tables 4, 7). Only two P2-P2′ sequences were repeated in different peptides and Y, L and F were present in 41%, 17.3% and 23.9% of all P1 positions (121) respectively. In the GluC library, of 141 peptides, 131 possessed different P2-P2′ sequences (Supplementary Tables 5, 7). Ten P2-P2′ sequences were repeated in different peptides and Y, L and F were present in 46%, 23.4% and 19.8% of all P1 positions (141) respectively. Notably, mKLK7 cleaved 10% and 40% of the active KLK7–cleaved tryptic (1,403) and GluC (353) peptides respectively.

### DmKLK7 is catalytically inactive

**Figure 3:**
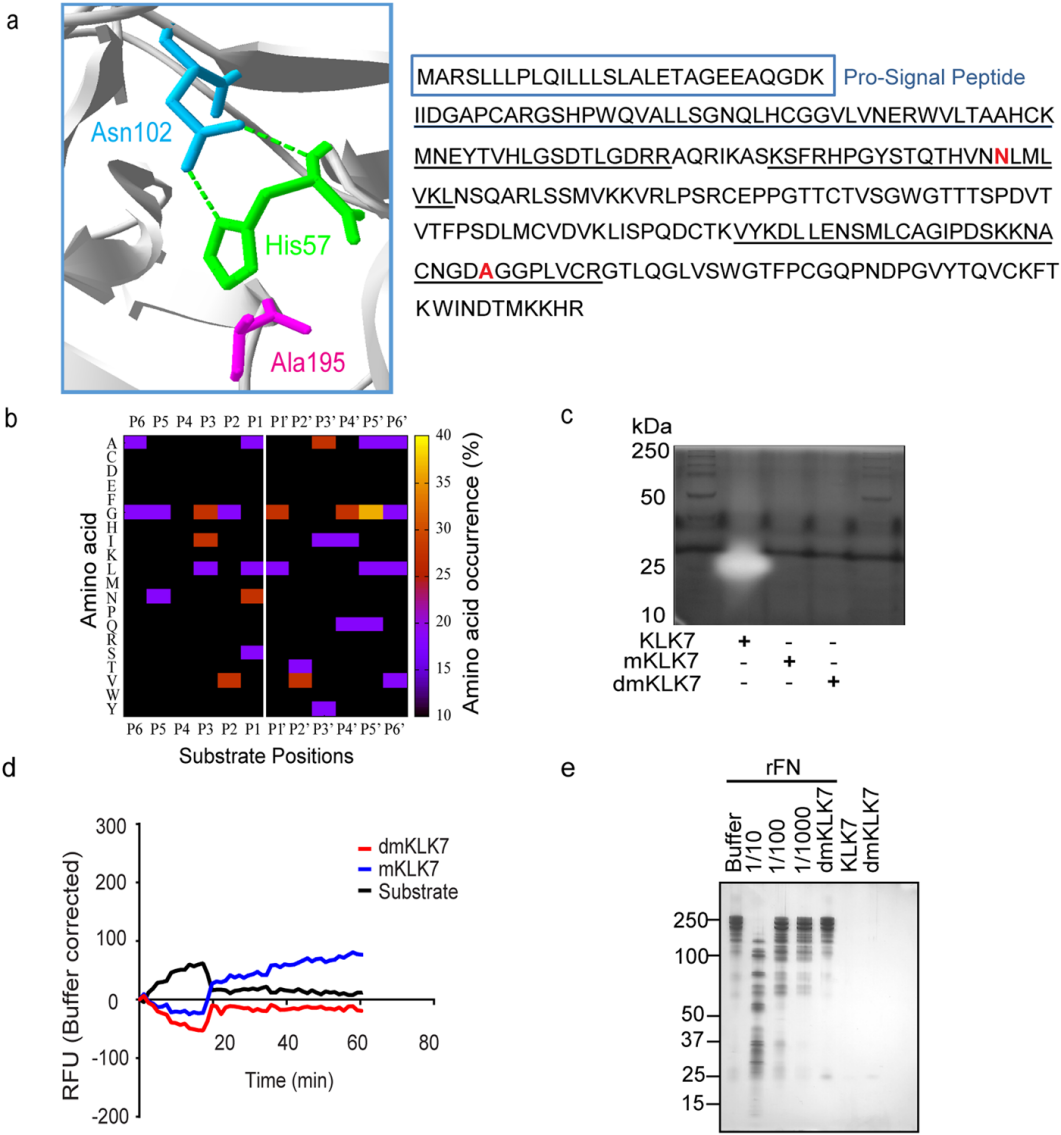
Activity profile of the double mutant KLK7 (dmKLK7). (**a**) Surface exposed residues corresponding to the catalytic triad of dmKLK7 (His57, Asn102 and Ala195) are shown on the 3D-structure available in PDB (accession: 2QXG.pdb in standard serine protease orientation) using SPDBV v4.10. Alongside, are the peptides identified in the MS-analysis (underlined) aligned with the KLK7 full length protein (UniProtKB; P49862-1). S195A and D102N mutations are as highlighted in red bold letters. (**b**) dmKLK7 cleavage site preferences in the tryptic-PICS library (1:50; enzyme/library) in the form of a 2D-heat map showing scattered cleavage specificities from P6-P6′ with no chymotryptic-like cleavage site specificities. (**c**) Casein-zymogram comparing active KLK7, mKLK7 and dmKLK7; (**d**) MeO-Suc-Arg-Pro-Tyr-MCA peptide substrate assay (n = 6, mean ± SD) with mKLK7 and dmKLK7; and (**e**) silver-stained SDS-PAGE with the protein substrate, FN, comparing active KLK (1/10 to 1/1000) with dmKLK7 .

Given the residual activity observed for the S195A mKLK7, we sought to mutate an additional residue in the catalytic triad to completely ablate activity. The dmKLK7 was produced in the *Pichia pastoris* expression system by mutating the D102N additional to the S195A mutation in the catalytic triad. Sequence coverage analysis of the produced dmKLK7 peptidase was performed using trypsin digestion followed by MS analysis. Only the peptides identified with high confidence (i.e. >99% protein and >95% peptide probability) were further analysed (Supplementary Table 1). The dmKLK7 was identified with 12 unique peptides, 22 unique spectra (of a total of 33) and 45% protein coverage (114/253 residues). Further, both the D102N and S195A mutations were detected in the tryptic peptides identified for dmKLK7 (Fig. 3a).

Interestingly, the dmKLK7-treated tryptic library resulted in 11 peptide identifications (background noise) with scattered cleavage site specificities within P6-P6′ (Fig. 3b). In fact, no L, F, and Y cleavages were observed at the P1 site as observed in the mKLK7-treated PICS libraries. Additionally, the P2-P2′ profiles in dmKLK7 showed no similarity to any of the KLK7 P2-P2′ profiles except for one sequence similar to the mKLK7-generated peptide (NQVALN^**↓**^PQNTV) in the tryptic library (Supplementary Tables 6, 7). Furthermore, no catalytic activity was observed on a casein-zymogram (Fig. 3c), with the peptide substrate (Fig. 3d), or with a protein substrate, fibronectin (Fig. 3e). Of note, dmKLK7 showed reduced catalytic activity compared to the ~1% residual activity of mKLK7 with the MeO-Suc-Arg-Pro-Tyr-MCA peptide substrate (Fig. 3d).

For further verification, Michaelis-Menten kinetics was calculated for KLK7, mKLK7 and dmKLK7 (Table 2, Supplementary Figure 4), which reflected the residual activity of mKLK7. The mKLK7 (S195A) showed a reduction in turnover number (k_cat_) by ~72-fold, whilst the dmKLK7 velocity values for increasing substrate concentrations did not fit the Michaelis-Menten kinetics curve, suggesting no residual activity of dmKLK7 (Supplementary Figure 4). S195A mutation caused a small increase in K_M_, ~1.5-fold, which may result from alterations in substrate binding upon S195A mutation as described for subtilisin mutants^19^. Interestingly, the enzyme efficiency dropped ~10^2^-fold compared to that of KLK7, although dmKLK7 resulted in complete inactivation. Mutation of both S195 and D102 residues in subtilisin and trypsin resulted in reduced turnover number (k_cat_)^19, 20^. To confirm negative contamination of mKLK7 at the DNA construct level, mKLK7 and dmKLK7 constructs were re-produced in the *Pichia* system. The newly produced mKLK7 also showed similar activity as observed previously (Supplementary Figure 5), while the newly produced dmKLK7 remained inactive (data not shown).

**Table 2:** Kinetics parameters of mutant KLKs (KLK4 and KLK7) at pH 8.0.

Enzyme	Active site configuration			k_cat_ (s^−1^)	K_M_ (µM)	k_cat_/K_M_ (s^−1^M^−1^)	% Activity*
KLK7	Ser195	Asp102	His57	24.5 ± 0.7	303.6 ± 20.16	8.08 × 10^4^	100
mKLK7	Ala195	Asp102	His57	0.34 ± 0.09	429.6 ± 224.2	7.93 × 10^2^	0.98
dmKLK7	Ala195	Asn102	His57	N/A	N/A	N/A	N/A
KLK4	Ser195	Asp102	His57	21.9 ± 0.6	40.53 ± 3.77	5.41 × 10^5^	100
mKLK4	Ala195	Asp102	His57	0.618 ± 0.09	101 ± 41	6.13 × 10^3^	1.13
dmKLK4	Ala195	Asn102	His57	N/A	N/A	N/A	N/A

Kinetic parameters for the active and mutant KLKs were measured using the MeO-Suc-Arg-Pro-Tyr-MCA (for KLK7) and d-VLR-AFC (for KLK4) substrates. K_M_ and k_cat_ values were calculated using the nonlinear regression analysis in Graphpad Prism (n = 6). Velocity values for dmKLK7 and dmKLK4 did not fit the Michaelis-Menten curve suggesting no residual activity and indicated as N/A. Data are presented as ±standard error. *Percentage activity of mKLKs is calculated relative to the catalytic activity of KLKs with MeO-Suc-Arg-Pro-Tyr-MCA or d-VLR-AFC substrates.

### Other KLK family members may have a similar propensity to retain catalytic activity with a single S195A mutation

**Figure 4:**
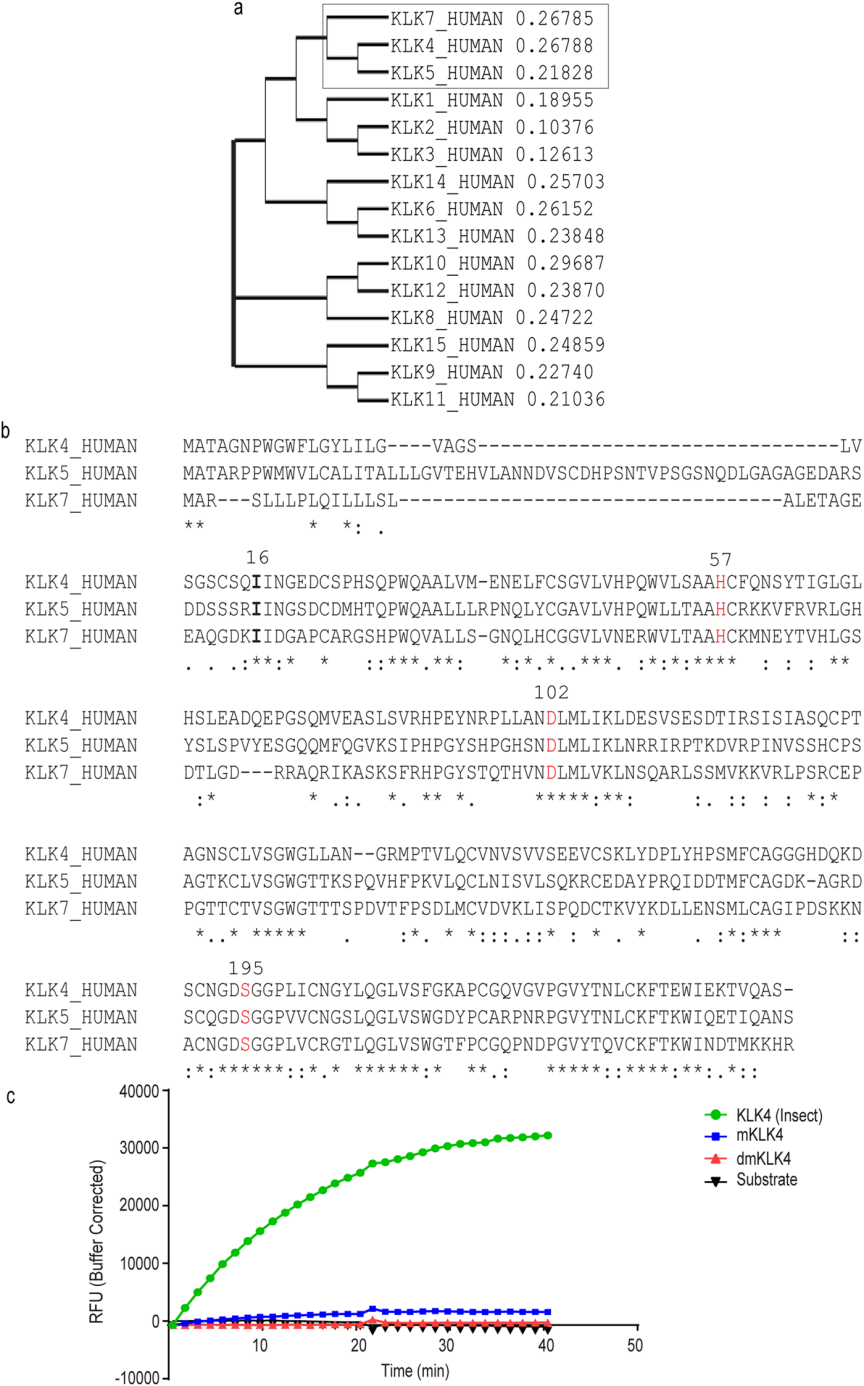
KLK family sequence similarity. (**a**) Human KLK full length protein sequences (UniProtKB) were aligned using Clustal Omega. Phylogram indicated high sequence similarity between KLK7, KLK4 and KLK5 (in box). (**b**) KLK4, 5 and 7 sequence alignment represents high sequence similarity adjacent to the catalytic triad residues (His57, Asp102 and Ser195 in red). N-termini of mature KLKs starting at Ile16 is shown in bold letters. “*”, a single, fully conserved residue; “:”, conservation between groups of strongly similar properties-scoring >0.5^; “ . ”, conservation between groups of weakly similar properties-scoring ≤0.5^; ^Gonnet PAM250 matrix. (**c**) mKLK4 showed residual activity against d-VLR-AFC peptide substrate compared to the dmKLK4.

The sequence similarity between the 15 members of KLK family is high^1^ with KLK7 showing highest sequence similarity to KLK4 and KLK5 (Fig. 4a) with particularly high sequence conservation surrounding the catalytic triad residues (Fig. 4b)^1, 21^. Thus, active KLK4, mutant KLK4 (S195A) and double mutant KLK4 (S195A and D102N) were produced. Interestingly, the mKLK4 showed partial activity by cleaving the peptide substrate, d-VLR-AFC (Fig. 4c, Supplementary Figure 4f), which was not observed with dmKLK4 (S195A/D102N) (Fig. 4c, Supplementary Figure 4g) and supported by kinetics values (Table 2). The mKLK4 showed 35-fold reduction in k_cat_ and a ~2.5-fold increase in K_M_ while the dmKLK4 kinetic values did not fit the Michaelis-Menten curve, suggesting no residual catalytic activity (Supplementary Figure 4d–g).

## Discussion

In the present study we report the first instance to analyse the prime and non-prime side specificities of KLK7 simultaneously using a MS-based approach. Moreover, we report the importance of extensive screening for the activity of mutated peptidases that are in use as catalytically inactive experimental controls due to detected residual activity of two Kallikrein-related peptidases, KLK4 and KLK7, mutated at the catalytic serine residues (S195A).

Herein, we used a mass spectrometry-based Proteomics Identification of protease Cleavage Sites (PICS) approach to determine cleavage preferences of KLK7 and mKLK7 (S195A). The active KLK7 cleavage site specificity profiles obtained in the present study are in excellent agreement with other studies where KLK7 was shown to have chymotryptic-like cleavage site specificities^9–11^. Of note, the cleavage preferences detected in this study are in accord with Skytt *et al*.^9^, where KLK7 was shown to cleave oxidized bovine insulin B chain predominantly C-terminal to Y, F and L, unlike other studies that have shown KLK7-mediated predominant cleavage of varying combinations, including F, Y or M^10^ and Y, A or M^11^. Despite significant differences in the starting material used, i.e. mammalian (C127 mouse) cell -derived KLK7 with the oxidized bovine insulin B chain as the substrate in Skytt *et al*.^9^, and *Pichia pastoris*-secreted KLK7 with HEK-293 cell-derived peptides in the current study, similar cleavage preferences were observed.

Moreover, in the present study, both trypsin- and GluC-derived libraries were used to emphasize the KLK7 cleavage site specificity profile. Tryptic peptides lack internal Lys and Arg residues and thus cannot be used in investigating subsite preferences for basic amino acids. Therefore, in addition to the tryptic library, PICS libraries were generated using GluC which cuts C-terminal to Glu and Asp. As a result, both the chymotryptic- and tryptic-like specificities of KLK7 were determined with high consistency using trypsin- and GluC-derived libraries respectively. Further, the use of different enzymes to prepare the peptide libraries overcame bias associated with peptide secondary structure formation and interactions that could occur distant to the active site cleft distorting cleavage events^22^. However, chemical modification of the sulfhydryls and primary amines of the peptides may have modified the peptidase cleavage preferences^13^.

Previous studies on KLK7 subsite preferences^9–11^ have employed synthetic peptide libraries of fixed length or oxidized bovine albumin B chain as a substrate, subsequently limiting the potential for subsite analysis. On the other hand, in the PICS assay, KLK7 peptidases were incubated with human proteome-derived highly diverse peptide sequence libraries of variable peptide length which can improve peptide recognition^22^. Moreover, these human proteome-derived peptides more closely represent physiological cleavage events due to similar glycosylation and other post-translational modifications, which may interfere with cleavage events, and were not accounted for in the previous studies. By detecting >1,000 individual cleavage sites in a single experiment, PICS allowed robust statistical analysis of subsite cooperation. Contrary to other studies, we were able to study the P6-P6′ range of KLK7 simultaneously, where other studies have studied P1-P4^10^ and P1′-P3′^11^ individually, thus lacking a more complete picture of the cooperativity between prime and non-prime side residues. Herein, we showed that KLK7 shows selectivity to hydrophobic amino acids in the P2-P2′ positions although hydrophilic K was preferred at the P1′ position.

*Pichia pastoris* is an efficient system for the secretion of correctly folded and N-linked glycosylated proteins, despite lacking the complex glycosylation that is present in higher order eukaryotes^23^. As shown in the literature, KLK7 and the mutants produced in the *Pichia* system were not N-linked glycosylated (data not shown) due to the presence of a sequon at Asn239 in the terminal α-helix^24^. Therefore, the observed high molecular weight bands of stratum corneum-extracted KLK7 may be partially O-linked glycosylated at Thr144 as predicted by the NetOGlyc 4.0 Server (http://www.cbs.dtu.dk/services/NetOGlyc/), however not further verified to date^25^. Although we cannot exclude that difference in such complex post-translational regulation may have had some effect on the cleavage preference of KLK7, we note the selectivity profiles obtained here were similar to those obtained in other studies using mammalian cell-line derived KLK7^9^. Further, whilst the kinetics values obtained here for KLK7 produced in *pichia* showed slightly different efficiency (k_cat_/K_M_ = 8.08 × 10^4^ s^−1^M^−1^) than reported for human stratum corneum-extracted KLK7 for the cleavage of the same MeO-Suc-Arg-Pro-Tyr-pNA/AMC substrate (k_cat_/K_M_ = 1.8 × 10^3^ s^−1^M^−1^), this may be the result of different detection approaches (absorbance vs fluorescence)^26^.

Identification of cleaved peptides in the mKLK7-treated library by PICS analysis revealed the residual proteolytic activity of mKLK7 that was not detected by the other methods used, such as casein-zymography, the chromogenic peptide substrate-based activity assay and a protein substrate, fibronectin (FN). These techniques employ a single recombinant protein digestion or a single peptide sequence/substrate (fluorescent/chromogenic substrate assays) which offer a limited number of potential cleavage sites that is efficiently cleaved by KLK7, but not by mKLK7. The PICS method on the other hand is a highly sensitive approach which provides proteome-derived peptide libraries with a multitude of potential cleavage sites for mKLK7^22^. Furthermore, cleavage products generated in the PICS assay can be sensitively detected by MS, whereas any cleavage of proteins into peptides by mKLK7 to this low extent would not be detected by less sensitive methods like zymography or silver staining. Interestingly, mKLK7 showed a slightly increased preference to Y at the P1 position compared to that of wild type KLK7. Substitution of S with A might have increased the hydrophobicity of the KLK7 substrate binding pocket, aiding the change of preferences observed in the PICS assay^27^. It is unknown to what extent this residual activity may have an effect in biological assays both *in vitro* and *in vivo* when analysing the role of KLK7. Hence it is important to be certain that a truly catalytically inactive control is used.

Given the findings above, a dmKLK7 was produced by mutating S195A and D102N in the catalytic triad in order to remove any residual activity of mKLK7. The dmKLK7 did not show any chymotryptic-like specificity with the PICS assay but only 11 peptide identities with scattered cleavage site specificities depicting background noise. Further, no activity was observed with the peptide substrate or the protein substrate, fibronectin. Moreover, the dmKLK7 kinetics values did not fit the Michaelis-Menten kinetics curve exhibiting no residual activity. Hence, the dmKLK7 (D102N/S195A) was considered catalytically inactive compared to the partially active mKLK7 (S195A).

KLK7 is a member of a family of 15 peptidases that are encoded by fifteen structurally similar conserved genes clustered together on chromosome 19q13.4^1, 28^. KLK7 showed high sequence similarity to KLK4 and KLK5 and high sequence conservation around the catalytic triad residues within these three genes. Thus, KLK4 and KLK5 may also require both point mutations, S195A and D102N in the catalytic triad to completely remove their catalytic activity. To scrutinize this possibility, we produced the wild-type KLK4 and mKLK4 with S195A mutation in the catalytic triad using the SF9 insect expression system. Use of different expression systems was expected to exclude any technical bias associated with individual expression systems and to ascertain that this residual activity is not an artefact of the expression system employed. Interestingly, the mKLK4 showed partial activity by cleaving the peptide substrate, d-VLR-AFC, which was not observed with dmKLK4 (S195A and D102N) and supported by kinetics values.

Observation of residual activity with both mKLK7 and mKLK4, produced independently of each other in two different expression systems confirmed that these KLKs are catalytically active (~1% activity of the wild-type) even in the absence of the serine nucleophile and is not an artefact of the expression system used. Notably our peptidases were prepared in isolation, preventing any possible contamination between active and mutant material, which could have been a possible confounder. This similar activity of a mutant lacking the conserved Ser195 residue in the catalytic triad, suggests a common additional enzymatic function may exist in this family, which could result from stabilization of a transition state by amino acids outside the catalytic triad^19^ or by substrate-assisted catalysis. In the latter possibility, substrate amino acids could directly participate in catalysis, such has been suggested previously in instances where one or more residues of the enzyme catalytic triad have been mutated^19, 29, 30^. Indeed, residual activity of S195A trypsin and subtilisin has been already reported^19, 20^, which could be similar to what we have observed in this analysis.

Additionally, in a previous study, on the related rat anionic trypsin, a 7-fold lower kcat was observed with S195T compared to S195A mutation, suggesting a further reduction in enzymatic activity by S195T substitution^20^. Further, it has been shown that D102N mutated trypsin can still bind to inhibitors directed at Ser195 or His57 several magnitudes slower than wild-type trypsin^31^, suggesting possible binding of the substrate to the Asp102 and/or His57 in the KLK7 catalytic triad upon S195A mutation. Perhaps such tetrahedral intermediates formed in the absence of the Ser195 could decompose by attack of a H_2_O OH^−^ ion. Thus, pH dependent measurement of mKLKs compared to their wild type KLKs would be of interest, especially given the possibility that the residual enzymatic activity of mKLK7 could be mediated by nucleophilic attack of a water OH^−^ ion. These instances highlight the highly cooperative role the catalytic triad plays in the catalytic mechanism and this is the first instance to report residual catalytic activity of S195A mutation of two members of the KLK family as already reported for other serine proteases, including trypsin and subtilisin^19, 20^. However, the alternative catalytic mechanism through which these serine peptidases function in the absence of Ser195 is yet to be elucidated.

## Conclusion

This is the first MS-based in-depth analysis to elucidate KLK7 subsite preferences and subsite co-operativity using human proteome-derived peptide libraries. Thereby, consistent with previous findings, KLK7 was found to exert chymotryptic-like cleavage preferences by cleaving predominantly C-terminal to Tyrosine, Leucine, Phenylalanine, Methionine and Tryptophan at the P1 position. KLK7 subsite preferences were also characterised in the prime and non-prime region simultaneously (P2-P2′), demonstrating a preference for hydrophobic residues in the non-prime and hydrophilic residues in the prime subsites. Moreover, we report residual catalytic activity of mKLK7 and mKLK4 with a single point mutation in the catalytic triad (S195A). Our observations suggest multiple point mutations (D102N/S195A), as validated herein, in the KLK7 or KLK4 catalytic triad to obtain a completely catalytically inactive KLK7 or KLK4 as experimental controls. Use of appropriate controls is important in deducing the precise roles of proteases and this information may aid in determining better controls for future experiments involving KLK7 or KLK4 in disease biology.

## Methods

### Recombinant KLK peptidase production

The pre-pro-KLK7 and KLK4 coding sequences [National Centre for Biotechnology Information (NCBI) reference sequence (RefSeq): NM_005046.3 and NM_004917.3 respectively] were PCR amplified from OVCAR-3 and LNCaP cells (ATTC) respectively. KLK4 pro-region was substituted with the enterokinase (EK) activation site (c. 1–69; NCBI RefSeq: NM_002769.4). S195A and D102N mutations were made using site-directed mutagenesis and sub-cloned into the pPIC9K vector followed by expression in *Pichia pastoris* GS115 cells^32^ or SF9 cells^33^ as described previously.

Secreted recombinant peptidases were purified from culture supernatant by cation exchange chromatography. Briefly, cells and insoluble debris were removed by centrifugation. Clarified culture supernatant was incubated with UNOSphere S cation exchange resin (Bio-Rad; 4 °C, O/N). Resin was recovered by column filtration and the KLK proteins were eluted using a stepped gradient of 50 mM K_2_HPO_4_ pH 7.5 buffer containing 0-500 mM NaCl, followed by a final elution at 1 M NaCl containing 50 mM K_2_HPO_4_. Fractions containing the KLK proteins were further purified using a pre-packed cation exchange Resource S column (GE Life Sciences), according to the manufacturers’ instructions. A 12,500:1 ratio of pro-KLK4: EK (*w/w*) was employed to process pro-KLK4 (23 °C, 18 h). EK-treated pro-KLK4 and pro-mKLK4 were further purified by anion exchange chromatography using a Resource Q column (GE Life Sciences) according to the manufacturers’ instructions.

To avoid cross-contamination, mKLKs were purified before wild type KLKs, and multiple blank runs carried out prior to, and following, purification of each peptidase. Fractions containing KLK peptidases, and no detectable contaminating proteins, were identified by silver-stained SDS-PAGE and pooled for further concentration with Amicon centrifugal filter units (Millipore, 3 kDa molecular weight cut off). Total protein concentration was determined using a BCA assay. Enzyme aliquots were stored at −80 °C and thawed aliquots re-frozen up to once only prior to use.

### Activity of the produced peptidases

#### Casein zymography

Native protein samples were resolved by a 12% SDS-PAGE gel incorporated with 1% casein. Gels were washed (Novex® renaturing buffer, Invitrogen), developed at 37 °C, O/N (Novex®), and stained with coomassie brilliant blue R-250.

#### Activity against a fluorogenic peptide substrate

Peptidases at 5 nM final concentration was incubated with a peptide substrate (MeO-Suc-Arg-Pro-Tyr-7-amino-4-methylcoumarin; 50 μM final) in assay buffer (0.1 M Tris-HCl; pH 8.0, 0.1 M NaCl, 10 mM CaCl_2_, 0.005% Triton X-100 [*v/v*], 37 °C). Relative fluorescence units (RFU) were recorded using the POLARstar® Omega Plate Reader Spectrophotometer (BMG Labtech; ex 400 nm, em 505 nm; 37 °C). The substrate with buffer was employed as the control. Results presented correspond to mean of RFU values +/− S.D. calculated on 6 technical replicates. Activity of KLK4 peptidases were measured using the d-Val-Leu-Arg-7-amido-4-trifluoromethyl coumarin (d-VLR-AFC) peptide substrate as above.

#### Active site titration

KLK7 was reacted (37 °C, 15 min) with serial dilutions of recombinant human (rh) Serpin A3/α1-Antichymotrypsin (ACT) (R&D Systems), a known covalent inhibitor of KLK7^34^, before fluorescent substrate addition (100 μM; Lys-His-Leu-Tyr-para nitroaniline or KHLY-pNA^35^) in assay buffer. The initial rate of KLK7 activity (Δ Optical Density min^−1^; Δ OD min^−1^), measured in a PolarStar Optima microplate reader (BMG Labtech; 405 nm; 37 °C), was plotted against respective inhibitor concentrations and extrapolated to Δ OD min-1 = 0 to find the concentration of ACT required for complete KLK7 inhibition, equal to the concentration of active KLK7 (1:1 stoichiometry). 2.5 nM ACT was considered required to completely inhibit 3 nM total KLK7 and considering the purity of ACT (90%), KLK7 was calculated to be composed of 75% active material [(2.5/3) * 90% = 75%]. Active site titration was performed to determine the concentration of active KLK4, with serial dilutions of α2-antiplasmin (R&D Systems), a known covalent inhibitor of KLK4^34^, and the d-Val-Leu-Arg-7-Amido-4-trifluoromethylcoumarin (d-VLR-AFC) trypsin-like peptide substrate (data not given) as for KLK7.

#### Michaelis-Menten Kinetics

Velocity (in relative fluorescence units [RFU]) of enzyme-substrate reaction was measured with increasing substrate (MeO-Suc-Arg-Pro-Tyr-7-amino-4-methylcoumarin [AMC]) concentration (0–250 μM) using the POLARstar® Omega Plate Reader Spectrophotometer (BMG Labtech; ex 400 nm, em 505 nm; 37 °C). RFU was converted to nM using a standard curve derived using a dilution series of AMC. Kinetics values (K_M_, k_cat_ and V_max_) were calculated using the non-linear regression analysis in the GraphPad Prism software. Results presented correspond to mean of RFU values +/− S.D. calculated on 6 technical replicates. D-VLR-AFC peptide substrate was used in determining KLK4 kinetics values.

### PICS specificity assay

#### Generation of PICS peptide libraries

The HEK293-F cell line (Invitrogen) was grown in suspension (5 L) using the chemically-defined, protein-free CD 293 medium (Invitrogen) supplemented with 4 mM GlutaMAX. Whole cell lysate was prepared as described previously^13, 22^. Briefly, the proteome derived from the HEK293-F cell line was digested with either trypsin or GluC (1:100; enzyme to proteome *w/w*) to generate respective peptide libraries (final peptide concentration of 1.3 μg μL^−1^) followed by LC-MS/MS analysis as described below in ‘PICS analysis of KLK7 and mKLK7’.

#### Determination of cleavage preferences

Experiment 1 contained the active KLK7 (5 μg total protein = 3.75 μg active enzyme, enzyme: library ratio 1:53) and the tryptic/GluC peptide library (200 μg). Buffer conditions were adjusted to 100 mM HEPES pH 7.4 and phosphate buffered saline (12 mM phosphate pH 7.4, 137 mM NaCl, 2.7 mM KCl) and incubated for 16 hrs, 37 °C. In experiment 2 and 3 the active KLK7 was replaced by either mKLK7 or dmKLK7 (5 μg total protein). In experiment 4 the peptide library was replaced by milliQ water. In experiment 5 the active KLK7 was replaced by milliQ water. Cleavage products were biotinylated and isolated using immobilised streptavidin. Peptides were purified using solid phase extraction (Strata-X 33 μm polymeric reversed phase 10 mg mL^−1^, Phenomenex). Results presented correspond to combined searches of cleavage sites/peptides identified in 2 technical replicates.

#### PICS analysis of KLK7 and mKLK7

Untreated as well as KLK7- and mKLK7-treated tryptic and GluC peptide libraries were analysed on a Prominence nano HPLC system (Shimadzu). Acidified sample aliquots were loaded onto a trap column (ReproSil pur C18-AQ 3 μm, 0.3 mm × 10 mm; Dr. Maisch, Ammerbuch-Entringen, Germany) and washed for 5 min at 30 μL min-1 using 100% eluent A. Peptide mixtures were subsequently flushed onto a capillary column (75 μm × 150 mm) packed in-house with ReproSil-Pur 120 C18-AQ 2.4 μm (Dr. Maisch) and separated at a flow rate of 300 nL min-1. Eluent A and sample loading eluent were 0.1% formic acid in 2% acetonitrile and eluent B was 0.1% formic acid in 80% acetonitrile. Peptides were separated at 50 °C using the following gradient: 0 min (5% eluent B) −5.5 min (5% B) −8.5 min (10% B) −90 min (30% B) −100 min (45% B) −108 min (95% B) −118 min (95% B) −120 min (5% B) −140 min (5% B).

Column-separated peptides were introduced either to an LTQ-Velos Pro (GluC peptide libraries) or an Elite (tryptic peptide libraries) Orbitrap mass spectrometer through a Nanospray Flex Ion Source (Thermo Fisher Scientific). Spray voltage was 1.5 kV with no sheath, sweep, or auxiliary gases used. The heated capillary temperature was set to 285 °C and the S-lens to 55%. The mass spectrometer was controlled using Xcalibur 2.2 software (Thermo Fisher Scientific) and operated in positive ion and data-dependent acquisition mode to automatically switch between Orbitrap-MS and ion trap-MS/MS. Full scan MS spectra (m/z 380–1700) were acquired in the Orbitrap with a resolving power set to 60,000 and 240,000 (at 400 m/z) for Velos Pro and Elite, respectively, after accumulation to a target value of 1 × 106 in the linear ion trap. The top 15 most intense ions with charge states ≥+2 were sequentially isolated with a target value of 5,000 and fragmented using rapid collision-induced dissociation (CID) scan mode in the linear ion trap. Fragmentation conditions were set as follows: 35% normalised collision energy; activation q of 0.25; 10 ms activation time; ion selection threshold 500 counts. Maximum ion injection times were 200 ms for survey full scans and 50 ms for MS/MS scans. Dynamic exclusion was set to 70 s. Lock mass of m/z 445.12 was applied with an abundance set at 0%.

#### PICS analysis of dmKLK7

Peptides of dmKLK7-treated tryptic peptide libraries were separated on a nano ACQUITY UPLC system (Waters, Milford, US), which enables the use of sub-2 μm particles resulting in narrower chromatographic peaks, improved signal-to-noise ratio and hence higher peak capacity and sensitivity. Digests were loaded onto a Symmetry C18 (5 μm, 180 μm × 20 mm; Waters) trap column, washed for 3 min at 15 μL min-1 using 2% eluent B and then separated on a BEH130 C18 column (1.7 μm, 75 μm × 200 mm; Waters) at a flow rate of 300 nL min-1. Eluent A was 0.1% formic acid in milliQ water and eluent B was 0.1% formic acid in acetonitrile. Peptides were separated at 35 °C using a sequence of linear gradients: starting from 5% B over 1 min, to 30% B over 90 min, to 45% B over 10 min and finally, to 95% B over 7 min, before holding the column at 95% B for a further 10 min. Column-separated peptides were electrosprayed into the LTQ-Orbitrap Velos Pro and analysed as above.

#### Data analysis

Tandem mass spectra were processed and searched through Proteome Discoverer 1.3 (Thermo Scientific) using the Mascot and SequestHT search engines. MS/MS spectra were searched against a SwissProt database with the taxonomy set to either ‘all entries’ (539,830 entries) or ‘Homo sapiens’ (20,307 entries, January 2013). For all database searches the following settings were kept constant: Precursor and fragment tolerance at ±20 ppm and ±0.8 Da, respectively. Oxidation of methionine and deamidation of glutamine and asparagine were set as variable modification. Depending on the experiment, parameters were modified: Enzyme specificity (semi-trypsin or semi-GluC) and maximum number of missed cleavages (2 or 3). Biotinylation of peptide N-termini, carbamidomethylation of cysteine and dimethylation of lysine either set to fixed or variable modifications. Scaffold (Proteome Software Inc., v4.0.5) was used to validate MS/MS-based peptide and protein identifications, and accepted where peptide and protein probabilities were >95.0% and >99.0%, respectively, to achieve an FDR <1.0% with at least 2 peptides.

## Electronic supplementary material

Supplementary Figures

Supplementary Tables
