# Supplementary material for: Mass spectrometry-based determination of Kallikrein-related peptidase 7 (KLK7) cleavage preferences and subsite dependency
Source: Sci Rep. 2017 Jul 28;7:6789. doi: 10.1038/s41598-017-06680-4 (PMC5533777; doi:10.1038/s41598-017-06680-4)
Supplement: Supplementary file 1 — Supplementary Figures [file 41598_2017_6680_MOESM1_ESM.pdf]

Mass spectrometry-based determination of Kallikrein-related peptidase 7 (KLK7) cleavage preferences and subsite dependency

Author names and affiliations:

Lakmali Munasinghage Silva[1,2], Thomas Stoll[3], Thomas Kryza[1], Carson Ryan Stephens[1], Marcus Lachlan Hastie[3], Helen Frances Irving-Rodgers[4], Ying Dong[1], Jeffrey John Gorman[3], Judith Ann Clements[1*]

[1]Queensland University of Technology (QUT), Institute of Health and Biomedical Innovation (IHBI) and School of Biomedical Sciences at the Translational Research Institute, 37 Kent Street, Woolloongabba, Queensland, 4102, Australia.

[2]Current Affiliation: Proteases and Tissue Remodelling Section, National Institute of Dental and Craniofacial Research, National Institutes of Science, 30 Convent Drive, Bethesda, Maryland 20892, USA.

[3]QIMR Berghofer Medical Research Institute, 300 Herston Road, Herston, Queensland, 4006, Australia.

[4]School of Medical Science, Griffith University Gold Coast Campus, Parklands Drive, Southport Queensland, 4215, Australia.

*Corresponding Author: Queensland University of Technology (QUT), Institute of Health and Biomedical Innovation (IHBI) and School of Biomedical Sciences at the Translational Research Institute, 37 Kent Street, Woolloongabba, Queensland, 4102, Australia.

j.clements@qut.edu.au

a

	P6	P5	P4	P3	P2	P1	P1prime	P2prime	P3prime	P4prime	P5prime	P6prime
A	0.7	0.8	0.9	1.2	1.1	0.2	1.7	**2.2**	1.4	1.2	1.4	1.3
C	0.8	1	1	1.5	1.2	0.7	1.2	1.2	0.7	1.1	0.7	1
D	0.6	0.8	0.5	0.9	1	0.1	0.7	0.3	1.5	1.3	1.7	1.7
E	0.6	0.8	0.4	1.1	0.8	0.1	0.7	0.4	1.3	1.2	1.3	1.3
F	0.8	0.8	0.9	0.6	0.5	**6**	0.5	0.7	0.4	0.4	0.4	0.4
G	0.8	0.9	1.1	1	0.6	0	1.8	1.5	1.6	1.8	1.3	1.5
H	0.4	0.7	0.8	0.8	0.5	0.8	1.2	0.9	1.4	1.5	1.3	1.6
I	0.6	0.9	1.1	0.9	1.2	0.1	0.5	0.9	0.9	1	1	1
K	0.1	0.1	0	0.1	0	0	0.2	0.2	0.2	0.2	0.2	0.3
L	0.5	0.7	0.9	0.5	1.6	**2.9**	0.7	0.9	0.8	0.8	0.8	0.8
M	0.6	1	0.5	0.9	0.9	**2.1**	0.6	0.7	0.5	0.7	0.8	0.5
N	0.7	0.9	0.8	2	0.7	0.7	1.2	1.4	1.3	1.4	1	0.8
P	1	0.9	1.6	0.6	1.7	0	0.2	1	1.4	1.5	1.5	1.5
Q	0.9	0.8	1.3	**2.1**	0.9	0.7	1.5	1	1.2	1.3	1.5	1.5
R	0	0.1	0	0	0	0	0.1	0.1	0.1	0	0	0
S	0.7	0.9	0.8	1.3	0.9	0.2	**2.7**	1.4	1.2	1.2	1.3	1.1
T	0.7	0.8	1	1.1	1.4	0.4	1.3	1.1	1	1	0.9	1.2
V	0.6	0.7	1.3	1.2	1.7	0.1	1.2	1.6	1	1.2	1.1	0.9
W	0.4	0.4	0.6	0.8	1	**3.8**	0.4	0.9	0.5	0.6	0.4	0.6
Y	0.6	0.7	0.8	0.8	0.9	**7.5**	0.2	0.4	0.2	0.3	0.2	0.1

b

	P6	P5	P4	P3	P2	P1	P1prime	P2prime	P3prime	P4prime	P5prime	P6prime
A	0.7	1	1	1.4	0.7	0.3	1.5	**2.2**	1.4	1.4	1.6	1.3
C	0.8	1.5	0.4	1.7	1.8	1.5	0.6	0.6	0.4	1	0.2	1.8
D	0.9	0.9	0.6	0.9	0.6	0.1	0.2	0.2	1.4	1.1	1.6	2.2
E	0	0	0	0	0	0	0.1	0	0	0.2	0	0.3
F	0.6	0.2	0.7	0.2	0.4	**3.9**	0.6	0.6	0.2	0.2	0.1	0.4
G	0.7	1.1	0.9	1	0.9	0.1	1.2	1.4	2	2.4	2	2.1
H	0.7	0.9	0.5	1.1	0.7	0.6	1.5	0.6	0.9	0.5	0.4	0.6
I	0.9	1.1	0.9	0.6	1	0	0.7	1.2	1.4	1	0.9	0.8
K	1.3	0.5	1.6	1.1	1	0.1	**3.5**	1.9	1.2	1	1.1	1.2
L	0.5	0.8	0.6	0.7	1.7	**2.7**	0.8	0.6	0.8	1.1	0.9	0.6
M	0.6	0.3	0.2	0.7	0.2	1.9	0.5	1.5	0.5	0.6	0.7	0.6
N	1	0.8	1	1.8	0.6	1.3	0.9	1	1	0.5	1.4	0.9
P	1.2	0.9	1.5	0.6	**2.4**	0	0	1.1	1.1	1.7	2.1	1.9
Q	0.6	0.9	1.4	1.7	1.1	1.5	1.1	1.1	1.3	1.1	1.6	1.1
R	0.5	0.5	1.3	0.6	0.4	1.2	1.3	0.4	0.4	0.4	0.1	0.1
S	0.6	0.7	0.8	1.6	1	0.2	1.4	1	1	0.7	0.9	0.9
T	0.5	0.8	0.6	0.8	0.9	0.4	0.7	0.6	0.9	1.2	1	0.8
V	0.5	1	1.4	1.1	1.8	0.1	1.1	1.7	1.6	1.1	0.9	1
W	0.3	0.3	0.6	0.6	0.6	**2.1**	0.3	0.6	0	0.6	0	0.6
Y	0.7	0.7	0.1	0.2	0.7	**7**	0.2	0	0.3	0.2	0.2	0.1

Supplementary Figure 1: KLK7 cleavage site specificities relative to natural abundance as calculated in CLIP PICS web server. a) Tryptic **b**) GluC PICS peptide libraries. Residues with an occurrence of more than 2 times the natural abundance (shaded in green) were considered non-random cleavage site preferences. KLK7 cleavage site specificities are predominantly distributed in P2-P2' in both the tryptic and GluC PICS libraries. However in P3, glutamine (Q) was preferred in the tryptic library, but not in the GluC library. In P2', alanine (A) was preferred in both libraries, confirming the importance of P2' site in determining KLK7 cleavage site specificities.

a

	P6	P5	P4	P3	P2	P1	P1prime	P2prime	P3prime	P4prime	P5prime	P6prime
A	5.6	6.4	7.3	9.7	9.3	1.5	**14.2**	**18.6**	11.5	9.6	11.2	10.6
C	1.1	1.4	1.3	2.1	1.6	0.9	1.7	1.6	1	1.5	0.9	1.3
D	3.3	4.1	2.6	4.8	5.6	0.4	3.8	1.8	8.2	7.1	9.3	9.4
E	4.3	5.1	3	7.1	5.5	0.9	4.6	2.9	8.6	8.1	9.1	8.8
F	2.9	3.2	3.6	2.5	1.9	**23.1**	1.8	2.8	1.4	1.5	1.4	1.6
G	5.4	6.3	7.8	6.9	4.6	0.3	**12.9**	**10.7**	11.6	12.6	9.3	10.9
H	0.8	1.5	1.9	1.9	1.1	1.9	2.7	2.1	3.2	3.4	3	3.7
I	3.6	5.4	6.8	5.1	7.3	0.4	2.8	5.3	5.6	5.8	5.8	5.8
K	0.3	0.5	0.1	0.6	0	0	1.2	1.2	1	0.9	0.9	1.8
L	5.3	6.3	8.9	5.1	**15.4**	**28.5**	6.5	8.6	8.1	7.6	7.7	8.1
M	1.4	2.4	1.3	2.1	2.1	**5**	1.5	1.8	1.3	1.6	2	1.1
N	2.9	3.7	3.4	8.3	3	2.7	5	5.8	5.2	5.6	4.2	3.3
P	4.8	4.2	7.7	2.6	7.8	0.2	0.9	4.6	6.6	7	7.1	6.9
Q	3.6	3.1	5.2	8.4	3.5	2.7	6	3.8	4.8	5	6.1	6.1
R	0	0.3	0	0.1	0	0	0.3	0.4	0.3	0.2	0.2	0.1
S	4.4	5.7	5.1	8.4	5.9	1.5	**17.8**	**9.1**	8.1	7.8	8.4	7.1
T	3.6	4.3	5.5	6.1	7.6	2.1	7.1	5.8	5.5	5.4	4.8	6.2
V	4.1	5.1	9	8.4	**12**	0.4	8.3	**11**	7	8	7.8	6
W	0.4	0.4	0.6	0.9	1.1	**4.1**	0.4	1	0.5	0.6	0.4	0.6
Y	1.8	2.1	2.2	2.4	2.5	**21.7**	0.6	1.1	0.5	0.8	0.6	0.4

b

	P6	P5	P4	P3	P2	P1	P1prime	P2prime	P3prime	P4prime	P5prime	P6prime
A	5.4	7.9	7.9	11.9	6.2	2.8	**12.2**	**18.1**	11.3	11.9	13	10.5
C	1.1	2	0.6	2.3	2.5	2	0.8	0.8	0.6	1.4	0.3	2.5
D	4.8	5.1	3.4	5.1	3.1	0.3	1.1	1.1	7.9	5.9	8.8	12.2
E	0.3	0.3	0	0	0	0	0.8	0	0.3	1.1	0.3	2.3
F	2.3	0.8	2.8	0.8	1.7	**15**	2.3	2.5	0.6	0.8	0.3	1.4
G	4.8	7.6	6.2	7.4	6.5	0.8	8.8	9.6	14.2	16.7	14.2	15
H	1.7	2	1.1	2.5	1.7	1.4	3.4	1.4	2	1.1	0.8	1.4
I	5.1	6.5	5.1	3.4	5.7	0	4	7.4	8.2	6.2	5.1	4.8
K	7.4	3.1	9.3	6.5	5.9	0.3	**20.7**	**11.3**	6.8	5.9	6.5	6.8
L	5.1	8.2	5.7	6.8	**16.4**	**25.8**	7.6	5.7	7.4	11	8.5	5.4
M	1.4	0.8	0.6	1.7	0.6	**4.5**	1.1	3.7	1.1	1.4	1.7	1.4
N	4.2	3.1	4	7.4	2.3	5.1	3.7	4.2	4.2	2	5.7	3.7
P	5.7	4	6.8	2.8	**11.3**	0	0	5.1	5.1	7.9	9.9	9.1
Q	2.5	3.4	5.7	6.8	4.2	5.9	4.5	4.2	5.1	4.5	6.2	4.2
R	2.8	2.8	7.1	3.4	2	**6.5**	7.4	2.3	2	2	0.8	0.8
S	4	4.8	5.4	10.2	6.5	1.4	9.3	6.8	6.5	4.5	5.7	5.9
T	2.8	4.5	3.4	4	4.8	2.3	3.7	3.4	4.8	6.2	5.4	4.5
V	3.4	7.1	9.3	7.6	**12.2**	0.8	7.6	**11.6**	11	7.9	6.2	7.1
W	0.3	0.3	0.6	0.6	0.6	2.3	0.3	0.6	0	0.6	0	0.6
Y	2	2	0.3	0.6	2	**20.4**	0.6	0	0.8	0.6	0.6	0.3

Supplementary Figure 2: KLK7 cleavage site specificities in percentages as calculated in CLIP-PICS web server. a) Tryptic **b**) GluC PICS peptide libraries. KLK7 cleavage site specificities are predominantly distributed in P2-P2' in both the tryptic and GluC PICS libraries. Residues with the highest preference are highlighted in red bold letters.

a

	P6	P5	P4	P3	P2	P1	P1prime	P2prime	P3prime	P4prime	P5prime	P6prime
A	0.4	0.4	0.9	1	0.5	0	2.2	2.9	1.7	1.3	1.7	2
C	0.6	0.6	1.2	0	0	0	1.2	2.4	0.6	0.6	1.2	0.6
D	0.3	0	0.1	0.5	0.1	0.1	0.5	0.3	0.8	1.1	1.4	1.5
E	0.5	0.9	0.3	0.7	0.7	0	0.6	0.1	0.9	0.9	1	1.1
F	0.6	0.9	1.3	0.6	1.3	6.2	0.9	1.1	1.1	1.5	0.9	0.6
G	0.9	0.4	1	0.5	0.2	0	1.5	1.3	1.2	1.2	0.9	1.6
H	0.4	0.7	1.1	1.1	0.4	0	1.8	1.5	0	1.5	0.4	1.5
I	1.1	0.7	1	1.5	1	0	0.4	0.6	0.7	1.1	0.7	0.8
K	0	0	0.1	0	0	0	0	0	0	0	0	0.1
L	0.9	0.9	0.8	0.8	2.2	1.8	0.5	0.9	0.6	0.9	1	0.7
M	0	2.7	1.4	0.3	0.3	3.4	1	0.7	0.3	1	0	0.7
N	0.4	0.2	0.4	2	0.6	1.2	1.6	1	1.8	1.6	1.6	1.4
P	0.9	1.2	0.7	0.2	1.6	0	0	1.2	1.8	1.4	1.6	1.6
Q	1	1.9	2.3	4.2	1	0.4	2.3	0.2	1	1.5	1.3	1.3
R	0	0	0	0	0	0	0.3	0.1	0.1	0	0	0
S	0.1	1	0.4	1	0.4	0	2.7	0.5	1.5	1.1	0.9	1.3
T	0.8	0.5	1.4	0.6	2.2	0	0.6	0.8	2.3	0.8	0.8	0.9
V	0.5	1	1.2	1.8	1.8	0	0.7	2.3	1.1	1.1	1.9	0.5
W	0	0.7	1.6	1.6	0.7	0	0	2.3	0	1.6	0	0.7
Y	1.1	0.6	0.6	0.3	2.3	14.2	0.9	1.1	0.6	0.9	1.1	0

b

	P6	P5	P4	P3	P2	P1	P1prime	P2prime	P3prime	P4prime	P5prime	P6prime
A	0.9	1.3	0.6	1.3	1.3	0.1	2.1	1.6	1.2	1	1.1	1.3
C	0.5	1	0.5	3.7	0.5	0	0.5	0.5	0	2.1	0.5	2.1
D	0.9	0.8	0	0.3	0.1	0	0.4	0	0.6	0.9	1.2	0.8
E	0	0	0	0	0	0	0	0	0	0	0.2	0.3
F	0.5	0.4	0.9	0	0.4	5.2	0.7	1.5	0.5	0.9	0.5	1.1
G	0.4	0.7	0.4	0.5	0.2	0	0.6	1.4	2.2	1.9	1.3	1.8
H	1.2	1.2	1.2	0	0	0	0.9	0.3	0.3	0	0.3	0.9
I	0.8	0.8	0.8	0.6	0.7	0	0.6	1.3	1.2	0.8	0.7	0.6
K	1.9	1.1	1.7	1	0.7	0	2.5	1.3	0.7	0.6	1.3	1.1
L	0.5	1	1.3	0.5	2.9	2.4	0.6	1	1.5	1.6	1.1	0.7
M	0.3	0	1.2	0.6	0.3	1.8	0.6	1.2	2.1	0.6	0.3	0.3
N	0.3	0.9	1.1	3	0.5	0.2	0.5	1.6	2.1	1.2	1.4	1.4
P	0.7	0.7	1.1	0.1	2	0	0	0.6	1.4	0.3	1.7	2.6
Q	1.3	0.9	1.1	2.2	1.1	0.5	1.8	1.1	1.3	1.8	1.6	0.9
R	0.8	0.8	1.5	0.8	0.1	0	0.9	0.3	0.3	0.4	0.3	0.3
S	0.4	0.4	0.8	1.6	0.9	0	1.7	1.4	1.1	0.7	1.1	1.1
T	0.1	0.8	0.9	1.6	0.8	0	1.9	0.8	0.4	1.2	1.6	0.8
V	1	1.1	1.5	1.2	2	0	0.9	1.9	0.9	1.9	1.3	1
W	0	0.6	0	0.6	1.3	0.6	0	0	0	0	0	0
Y	0.5	1	0.5	0.7	0.7	15.8	1	0.2	0.2	0.7	0.2	0.7

Supplementary Figure 3: mKLK7 cleavage site specificities relative to natural abundance. In **a**) tryptic **b**) GluC PICS-peptide libraries residues with an occurrence of more than 2 times the natural abundance (shaded in green) were considered non-random cleavage site preferences. mKLK7 cleavage site specificities are predominantly distributed in P2-P2' in both the tryptic and GluC PICS libraries. However in P5, methionine (M) and in P4 glutamine (Q) were also preferred in the tryptic library, but not in the GluC library whereas glutamine (Q) was preferred in both libraries at the P3 position.

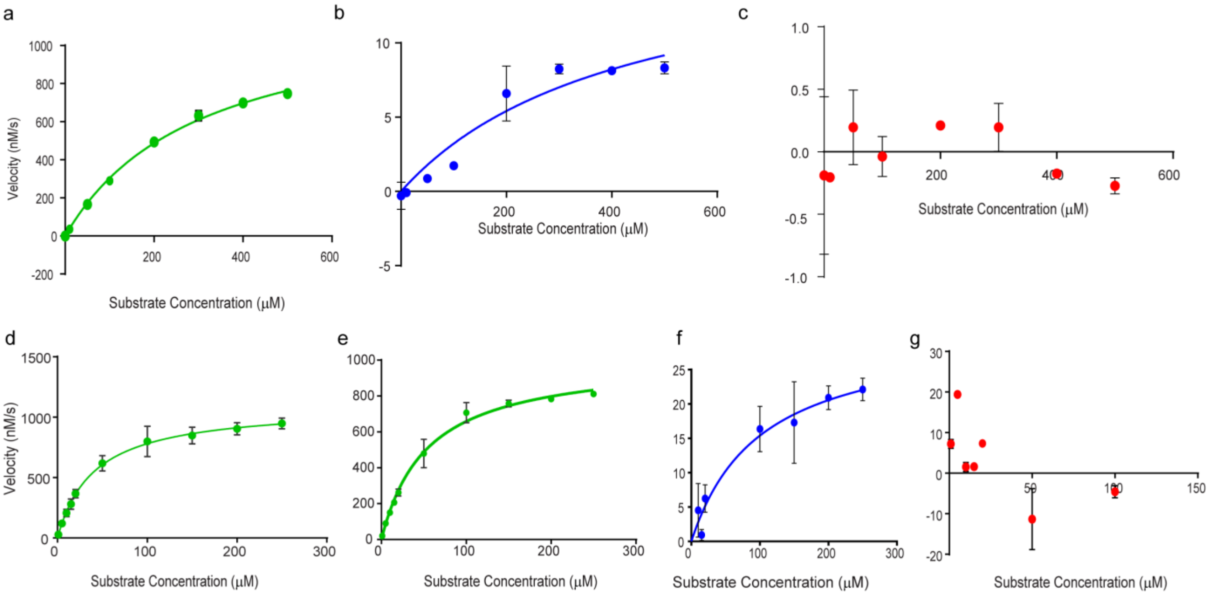

Supplementary Figure 4: Michaelis-Menten kinetics curves for KLK7 and KLK4 peptidases. **a**) KLK7 and **b**) mKLK7 velocity values against substrate (MeO-Suc-Arg-Pro-Tyr-MCA) concentration fitted the Michaelis-Menten curve but not **c**) dmKLK7. All KLK7 peptidases were produced in the yeast *Pichia pastoris* expression system. **d**) KLK4 produced in the insect and **e**) yeast expression systems displayed similar V_{max}, K_M and K_{cat} values, and thus suggesting similar activity of these peptidases produced in two different expression systems. **f**) The mKLK4, produced in the insect SF9 system, fitted the Michaelis-Menten curve, but not **g**) dmKLK4 as in **c**) dmKLK7. mKLK4 and dmKLK4 were produced in the insect SF9 system and yeast *Pichia pastoris* expression system, respectively. Results displayed are the mean ± SD based on N = 6; *Error bars are not visible when the error bars are shorter than the height of the symbol.

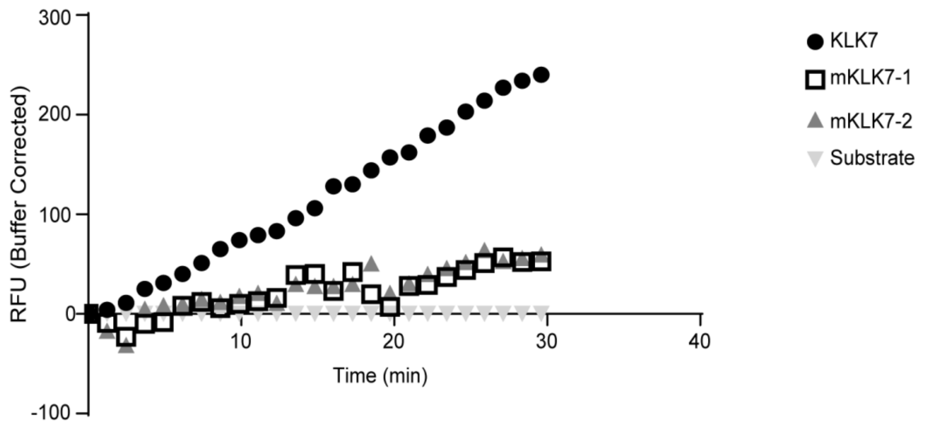

Supplementary Figure 5: Activity of two batches of mKLK7 produced independently compared to KLK7. KLK7 (green triangles); mKLK7-1 (batch1; dark blue filled circles) and mKLK7-2 (batch 2; pale blue outlined squares) activity with a KLK7-specific peptide substrate MeO-Suc-Arg-Pro-Tyr-MCA: time versus relative fluorescence units (RFU; corrected mean±SD) plot showing activity of mKLK7-1 and mKLK7-2 compared to KLK7 (n=3; mean±SD).
